# A bio-inspired microwave wireless system for constituting passive and maintenance-free IoT networks

**DOI:** 10.1093/nsr/nwae435

**Published:** 2024-12-09

**Authors:** Buyun Yu, Hong-Qin Wang, Lu Ju, Ke-Xin Hou, Zhi-Da Xiao, Jun-Lin Zhan, Chao Zhang, Hao Chen, Binghao Wang, Zhen-Guo Liu, Ying-Shi Guan, Cheng-Hui Li, Tie Jun Cui, Wei-Bing Lu

**Affiliations:** State Key Laboratory of Millimeter Waves, School of Information Science and Engineering, Southeast University, Nanjing 210096, China; Center for Flexible RF Technology, Frontiers Science Center for Mobile Information Communication and Security, Southeast University, Nanjing 210096, China; State Key Laboratory of Coordination Chemistry, School of Chemistry and Chemical Engineering, Collaborative Innovation Center of Advanced Microstructures, Nanjing University, Nanjing 210023, China; State Key Laboratory of Millimeter Waves, School of Information Science and Engineering, Southeast University, Nanjing 210096, China; Center for Flexible RF Technology, Frontiers Science Center for Mobile Information Communication and Security, Southeast University, Nanjing 210096, China; State Key Laboratory of Coordination Chemistry, School of Chemistry and Chemical Engineering, Collaborative Innovation Center of Advanced Microstructures, Nanjing University, Nanjing 210023, China; State Key Laboratory of Millimeter Waves, School of Information Science and Engineering, Southeast University, Nanjing 210096, China; Center for Flexible RF Technology, Frontiers Science Center for Mobile Information Communication and Security, Southeast University, Nanjing 210096, China; State Key Laboratory of Millimeter Waves, School of Information Science and Engineering, Southeast University, Nanjing 210096, China; Center for Flexible RF Technology, Frontiers Science Center for Mobile Information Communication and Security, Southeast University, Nanjing 210096, China; State Key Laboratory of Millimeter Waves, School of Information Science and Engineering, Southeast University, Nanjing 210096, China; Center for Flexible RF Technology, Frontiers Science Center for Mobile Information Communication and Security, Southeast University, Nanjing 210096, China; State Key Laboratory of Millimeter Waves, School of Information Science and Engineering, Southeast University, Nanjing 210096, China; Center for Flexible RF Technology, Frontiers Science Center for Mobile Information Communication and Security, Southeast University, Nanjing 210096, China; School of Electronic Science and Engineering, Southeast University, Nanjing 210096, China; State Key Laboratory of Millimeter Waves, School of Information Science and Engineering, Southeast University, Nanjing 210096, China; Center for Flexible RF Technology, Frontiers Science Center for Mobile Information Communication and Security, Southeast University, Nanjing 210096, China; School of Chemistry and Chemical Engineering, Southeast University, Nanjing 210096, China; State Key Laboratory of Coordination Chemistry, School of Chemistry and Chemical Engineering, Collaborative Innovation Center of Advanced Microstructures, Nanjing University, Nanjing 210023, China; State Key Laboratory of Millimeter Waves, School of Information Science and Engineering, Southeast University, Nanjing 210096, China; State Key Laboratory of Millimeter Waves, School of Information Science and Engineering, Southeast University, Nanjing 210096, China; Center for Flexible RF Technology, Frontiers Science Center for Mobile Information Communication and Security, Southeast University, Nanjing 210096, China

**Keywords:** flexible microwave system, biomimetic design, responsive material, backscatter communication, hybrid energy harvesting, self-healable polymer

## Abstract

With the rapid expansion of wireless networks, the deployment and long-term maintenance of distributed microwave terminals have become increasingly challenging. To address these issues, we present a bio-inspired microwave system to constitute passive and maintenance-free wireless networks. Drawing inspiration from vertebrate skeletons and skins, we employ stimuli-responsive polymer with tunable stiffness to support and protect sensitive electromagnetic structures, and synthesize self-healable skin-like polymer for system encapsulation. Owing to the biomimetic strategy, our system combines outstanding flexibility, electromagnetic stability, structural robustness, and self-healable performance. On the other hand, to address power supply issues, our system modulates ambient electromagnetic waves to achieve long-range wireless communication, and the hybrid energy harvesting strategy allows the system to capture energy from ambient light and microwaves, thereby eliminating the need for batteries or power cables. Multidisciplinary innovation enables our system to be deployed almost anywhere and supports stable, battery-less, and maintenance-free wireless communication.

## Teaser

This article presents a bio-inspired microwave system with stiffness-tunable ability, hybrid-energy harvesting, low-power backscatter communication and self-healable encapsulation, enabling battery-free wireless sensing across diverse conditions and harsh environments.

## INTRODUCTION

Next-generation Internet of Things (IoT) and 6th-generation (6G) mobile networks necessitate the ubiquitous deployment of microwave wireless systems to support seamless connections [1–4]. These microwave terminals can enhance the network's coverage and perceptual ability, fostering communication between humans, smart devices, and the physical world [5–13]. However, as the network scale continues to grow, the massive distributed systems have resulted in an exponential increase in deployment difficulty and maintenance costs. Therefore, to ensure the sustainable advancement of future networks, there is an urgent desire for a new-generation microwave wireless system that combines features of broad compatibility, superior durability, and battery-free operation [14,15].

In response to the demands of ubiquitous connectivity, researchers have developed a series of advanced flexible microwave systems capable of seamlessly deploying onto diverse irregular objects to facilitate wireless communications [16–20]. These soft systems demonstrate much better conformability than conventional rigid electronics, showcasing tremendous potential in wearable applications. However, the use of soft microwave systems for outdoor wireless network expansions or off-body applications presents challenges in reliability and long-term maintenance due to their inability to withstand high contact forces, loads, and mechanical damage [21]. To address these inherent limitations, researchers have begun to employ stimuli-responsive materials with tunable stiffness to create transformative electronic systems (TESs) [21–25]. This strategy promises to overcome the natural contradiction between flexibility and robustness, but significant challenges remain in implementing microwave wireless systems on responsive materials. Unlike low-frequency electronics, microwave systems operate in the radio-frequency (RF) range, making them highly sensitive to material characteristics, structural stability, and mechanical damage. Current TESs have not taken into account the structural variations and mechanical damage caused by repeated contact forces and deformations, hampering the prolonged stability and maintenance of microwave wireless terminals [26,27]. Besides, energy supply is another critical bottleneck for IoT and distributed mobile networks. Despite the fact that state-of-the-art TESs can support wireless data transmission, they still depend on power supply cables for continuous operation [25]. The lack of innovation in environmental energy harvesting strategies and low-power wireless communication restricts the potential applications of TESs in future IoT networks. These scientific issues are interrelated, and single-disciplinary innovation is insufficient to simultaneously overcome these challenges. Up till the present moment, there remains an uncharted terrain for exploring a microwave wireless system that overcomes the critical obstacles faced by next-generation IoT networks.

**Figure 1.**
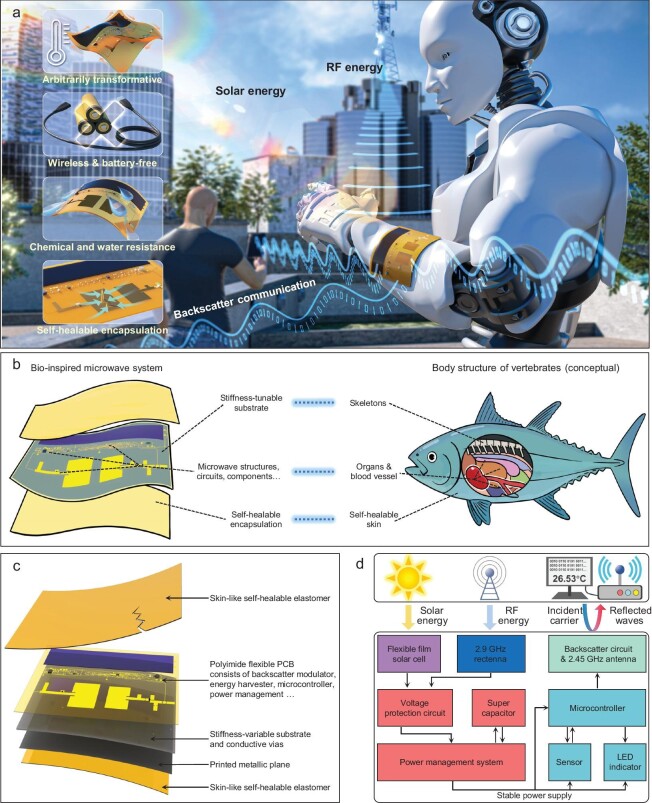
Concept illustration and operating principle of the bio-inspired wireless communication and energy harvesting system. (a) The future application illustration of the system. (b) Conceptional illustration of the bio-inspired strategy. (c) The exploded-view illustration of the constituent layers. (d) The operational diagram of the entire system.

In this paper, we merge the bio-inspired design strategy with electromagnetic sciences to address the aforementioned scientific challenges. Inspired by the body structure of vertebrates, we propose a biomimetic strategy to create microwave systems for distributed IoT and wireless communication networks. To imitate the properties of vertebrate skeletons, we overcome technical barriers in manufacturing large-scale, high-performance microwave wireless systems on mechanically tunable responsive materials to support and protect fragile electromagnetic structures and circuits. Furthermore, inspired by animal skins, we synthesize a skin-like soft polymer with self-healable properties, excellent strength, and chemical stability, which provides a durable encapsulation for the proposed system against contact force, mechanical damage, or liquid corrosion [28–30]. This biomimetic design strategy endows the proposed electromagnetic system with outstanding reliability, durability, and broad utility. In electromagnetic sciences, we incorporate the ultra-low-power backscatter communication scheme into the system configuration, allowing the bio-inspired system to modulate and reflect ambient electromagnetic waves for wireless communications, rather than generating electromagnetic waves like conventional systems. As a result, the power consumption of our system diminishes to a mere 2.38% of that of conventional modules. Building on this, we devise an efficient solar-microwave hybrid energy harvester and intelligent power management scheme to capture environmental electromagnetic energy. By modulating and harvesting ambient electromagnetic waves, our system enables long-range wireless communication and sensing without relying on any batteries or power cables. Compared with previous TESs, the proposed system achieves significant advancements in electromagnetic stability, battery-free operation feature, maintenance complexity, and long-term sustainability. As shown in Fig. 1a, with these unique characteristics, the bio-inspired microwave system can be deployed in many scenarios to support stable, battery-less, and maintenance-free IoT networks, paving the way for the future of ubiquitous wireless connectivity.

## RESULTS

### Design concepts and operational principle

Unlike low-frequency electronic devices, microwave systems operate in the Gigahertz range, where their electromagnetic performance is sensitive to structural variations, contact forces and damages. In this regard, microwave devices are similar to organs, and they are equally intricate and sensitive. For vertebrates, skeletons provide excellent protection for delicate internal organs, while sophisticated joints ensure the flexibility and agility of the body. Additionally, skins and muscles shield organs from contact forces and mechanical damage, and they can self-repair after injury. Drawing inspiration from the body structure of vertebrates, as shown in Fig. 1b, we create a large-scale microwave system on thermal-stimulus responsive microwave material with tunable stiffness to support and protect sensitive microwave components, and encapsulate the whole system in a self-healable soft encapsulation. The bio-inspired microwave system exhibits outstanding reliability and electromagnetic stability at room temperature, while rapidly transitioning to the flexible state when heated, thereby combining exceptional robustness and flexibility [31–33]. The soft skin-like encapsulation protects fragile electromagnetic components from contact force and damage, while the self-healable feature significantly enhances the system's longevity and environmental stability. Through the innovative biomimetic strategy, this kind of transformative microwave system demonstrates exceptional endurance and electromagnetic stability, along with flexibility approaching that of natural flexible systems.

Fig. 1c–d show the exploded-view illustration and operational principles of our bio-inspired wireless communication and energy harvesting (BWCEH) system. A 2.45 GHz (ISM band) backscatter modulator takes responsibility for wireless communication. A backscatter communication scheme achieves wireless data transmission by modulating electromagnetic waves in environments, obviating the need for generating electromagnetic wave energy. This approach significantly decreases system power consumption by an order of magnitude or more, and lays the foundation for the realization of a battery-free system. At the same time, we designed an efficient solar-microwave hybrid energy harvesting-management system, constantly capturing electromagnetic energy and providing a stable power supply for the proposed BWCEH system. The cooperation of ultra-low power consumption and efficient hybrid energy harvesting technology enables the BWCEH system to operate steadily without the need for any replaceable lithium batteries or power cables in various environments, thus addressing both the power supply and maintenance issue.

### Responsive microwave substrate with tunable stiffness

**Figure 2.**
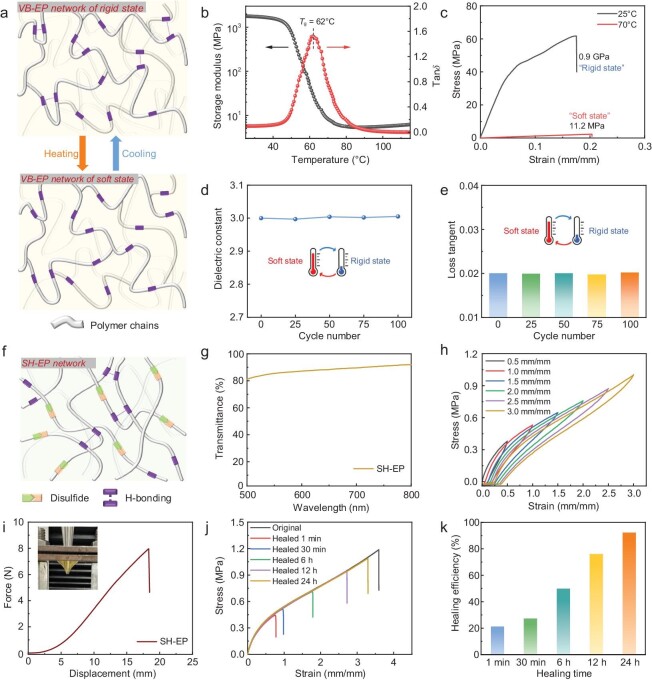
Electronic materials designed for the bio-inspired microwave system. (a) Schematic illustration of the crosslinking networks for VB-EP. (b) Temperature-sweep DMA result of VB-EP. (c) Stress-strain curves of VB-EP at 25°C and 80°C, showing the behavior of variable stiffness. (d) Dielectric constant of the VB-EP material after heating-cooling cycles. (e) Loss tangent of the VB-EP material after heating-cooling cycles. (f) Schematic illustration of the dynamic crosslinking networks for SH-EP. (g) UV-vis transmission spectrum of the SH-EP film (thickness ≈ 500 µm). (h) Cyclic stretching-releasing curves of SH-EP from 50% to 300% without relaxing time (tensile speed = 50 mm min^−1^). (i) Puncture curves for SH-EP under the deformation rate of 50 mm min^−1^ (the thickness of the film is 0.6 mm). (j) Stress-strain curves of the original and self-healed SH-EP films after different healing times at 25°C. (k) The self-healing efficiencies of SH-EP films for different durations at 25°C.

Substrate material, acting as the ‘bones’ to support sensitive microwave structures, directly determines the electromagnetic performance, structural reliability, and manufacturing difficulty of high-frequency systems. To achieve our goals, we aim for a substrate material that not only possesses good ‘flexibility’ to adapt to different surfaces but also exhibits excellent ‘rigidity’ to protect sensitive microwave systems. Additionally, it should have low production costs and a straightforward synthesis method to meet the requirements of mass production. The epoxy compounds contain polar structures such as ether bonds and hydroxyl groups, thus imparting good toughness and adhesion to the polymers [34,35]. We have chosen bisphenol A-based epoxy resin as the backbone of the polymer due to its good mechanical strength, rigidity and low cost [36,37]. By blending bisphenol A-based epoxy (GELR-128) with ether bond-rich poly(propylene glycol) bis(2-aminopropyl ether) (D230) and subsequent treatment at 80°C for 2 h, three-dimensional (3D) crosslinked variable stiffness materials can be obtained (denoted as VB-EP, Fig. 2a, Figs S1–S2). The thermo-mechanical properties of VB-EP are tested using a dynamic mechanical analyzer (DMA) from 25°C to 115°C at a rate of 3°C min^−1^ (Fig. 2b). The storage modulus of VB-EP is as high as 1.77 GPa at room temperature and gradually decreases to ∼10 MPa (>70°C) as the temperature increases, due to the glass transition from the glassy to the rubbery state. The glass transition temperature (*T*_g_), according to the peak of tan*δ* is 62°C. The temperature-dependent stress-strain curves also show that VB-EP possesses excellent rigidity at room temperature and has a Young's modulus as high as 0.9 GPa, and its stiffness will decrease sharply to 11.2 MPa when the temperature is increased to 70°C (Fig. 2c), which is in agreement with the phenomenon of stiffness variation obtained from the DMA test (Fig. 2b). According to the thermal gravimetric analysis (TGA) data (Fig. S3), the polymer is stable below 330°C, thus ensuring the material’s stability and facilitating the integration of the system (even low-temperature welding can still bring temporary high temperatures of ∼180°C).

In addition to mechanical performance, electromagnetic characteristic is also a core indicator of microwave materials. To obtain the electromagnetic properties of the VB-EP, we measured a 1 mm-thick VB-EP prototype in a split post dielectric resonator. Experimental results demonstrate that the material has a dielectric constant of 3.0 and a loss tangent of ∼0.02 (at 5.1 GHz). Appropriate dielectric properties of VB-EP material will ensure the electromagnetic performance of microwave structures. Since the mechanical tunability depends on the thermal stimuli, the electromagnetic characteristics of the VB-EP material should possess good thermal stability. For VB-EP materials, the phase transition (around *T_g_*) and the breaking and recombination of hydrogen bonds during the heating and cooling process are reversible, which is illustrated in Fig. 2a. Consequently, this responsive polymer possesses completely reversible and stable mechanical and electromagnetic properties. We conducted cyclic heating-cooling experiments on the VB-EP material and measured its electromagnetic characteristics. Experimental results reveal that even after multiple heating-cooling cycles, the electromagnetic characteristics of the VB-EP material remain virtually unchanged (Fig. 2d–e). Therefore, transformative microwave devices based on VB-EP substrates can undergo repeated deformations while maintaining consistent electromagnetic performance. In addition to remarkable physical and electromagnetic properties, the synthesis of this material is straightforward and the raw materials are cost-effective, which is advantageous for large-scale production.

### Self-healable elastomer acts as the skin of the microwave system

In addition to a reliable substrate material, as the ‘skin’ of electronic systems, soft encapsulation can shield fragile microwave structures against contact force and mechanical damage. Like our skins, ideal encapsulants are expected to exhibit excellent elasticity, mechanical strength, and self-healing ability [38]. In order to obtain self-healable and stretchable encapsulants at room temperature, we introduce aromatic disulfide bonds (4-Aminophenyl disulfide) with higher reversibility into the polymer [39,40], and use flexible thiol-terminated liquid polysulfide oligomer (LP3) and epoxy-terminated polydimethylsiloxane (epoxy-PDMS) as the backbones to enhance the mobility of the polymer chains, which is conducive to self-healing and ensuring the polymer's stretchability at room temperature. We obtained the 3D crosslinked polymer (denoted as SH-EP), which is able to improve the elasticity, from the reaction between amino/thiol and epoxy groups (Fig. 2f and Fig. S4). Meanwhile, epoxy resins are commercially available and the curing process is solvent-free. SH-EP is characterized by FT-IR spectroscopy (Fig. S5). Fig. 2g shows that the transmittance of SH-EP in the visible wavelength range of 500–800 nm is >80% for a 0.5 mm-thick film with high transparency, which is beneficial for the visual monitoring of the system's operating status and the transmission of sunlight for energy harvesting. In addition, SH-EP exhibits excellent elasticity and low hysteresis (Fig. 2h), which may be attributed to the 3D crosslinked structure that can maintain the integrity of the network even if some of the disulfide bonds are disrupted during stretching [41]. SH-EP also exhibits good puncture-resistant behavior with a maximum load of 8 N at a puncture speed of 50 mm^−1^ (Fig. 2i). The puncture energy is calculated to be 59.84 mJ [42]. SH-EP maintains an elastic state in the temperature region from 0°C to 120°C and the modulus during heating and cooling is highly coincident, indicating good thermal stability as well as reversibility (Fig. S6). According to the TGA data, SH-EP is stable below 278°C (Fig. S7). The SH-EP films, when cut into two separate pieces and contacted for 24 h at room temperature, can achieve a self-healing efficiency of 92.17% and can be stretched up to 200% of the original sample (Fig. 2j, k and Fig. S8). As shown in Fig. S9, the measured low *T*_g_ of SH-EP (−41°C) enables them to have a highly elastic state and have strong polymer chain mobility at room temperature, which could facilitate self-healing [43]. To further understand the dynamic properties of this crosslinked network, temperature-dependent stress relaxation experiments are performed (Fig. S10). The average apparent activation energy (*E*_a_) for chain mobility is calculated to be 55.74 kJ mol^−1^ [44], which may be due to the rapid exchange of dynamic disulfide bonds and hydrogen bonds. The photographs of the synthetic VB-EP substrate and SH-EP film are shown in Fig. S11, and these electronic materials will ensure the electromagnetic performance, transformative ability, and reliability of the microwave system.

### Manufacturing strategy, transformative ability and superb endurance

**Figure 3.**
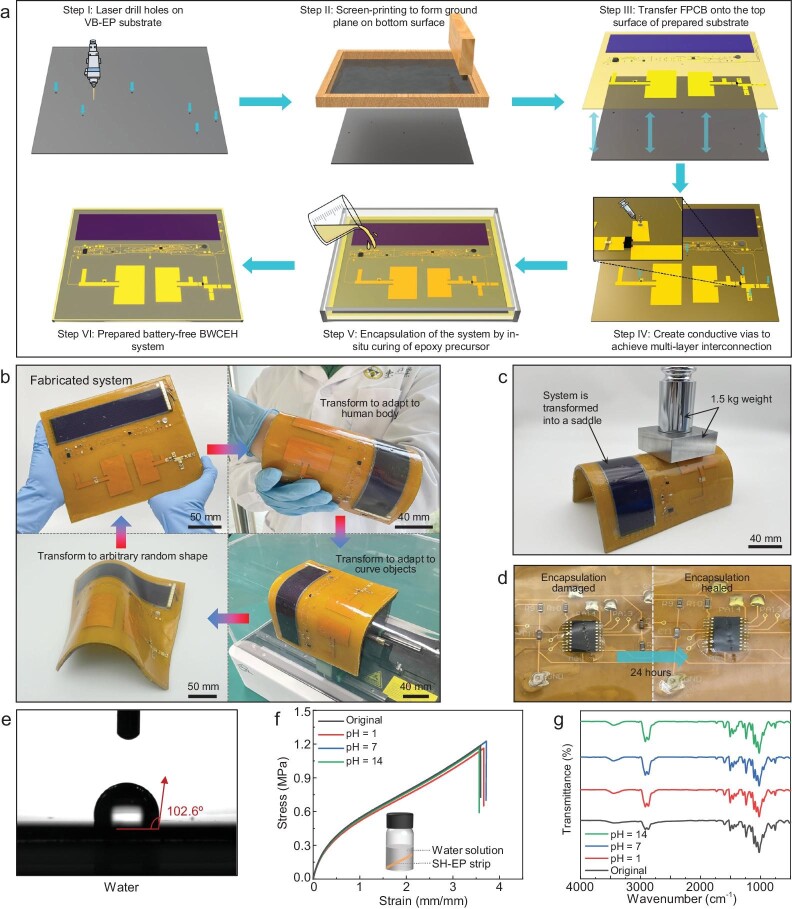
Manufacturing strategy, fabricated system and endurance demonstration. (a) The manufacturing processes. (b) The fabricated system and transformation ability demonstration. (c) Mechanical strength demonstration of the BWCEH system. (d) Self-healing progress of the SH-EP encapsulation. (e) Surface wettability of SH-EP encapsulation, indicating excellent water resistance performance. (f) Tensile stress-strain curves of SH-EP encapsulation after being immersed in solution with different pH for 48 h. (g) FT-IR spectra of SH-EP encapsulation at different conditions. Tensile stress-strain and FT-IR spectra measurements prove that our system possesses the ability to resist strong acidic and alkaline environments.

For microwave systems, manufacturing processes profoundly influence structural stability and electromagnetic reliability. To support stable electromagnetic performance on irregular and unstable objects, we have devised a method to manufacture large-scale, multi-layer and high-performance microwave systems on responsive substrates, which is illustrated in Fig. 3a. We manufactured microwave structures along with digital circuits on the polyimide film, and integrated most of the electronic components onto the flexible circuit by the surface mount technology (SMT) process, which is shown in Fig. S12. Next, we utilized the electronic design automation (EDA) software and high-precision laser cutting machine to drill holes in the 2-mm thick VB-EP microwave substrate, laying the groundwork for multilayer interconnection (Fig. S13). Then, we deposited a layer of highly conductive metallic film on the bottom surface of the VB-EP material, serving as the ground plane for microwave structures. After that, we spin-coated a layer of adhesive on the upper surface of the substrate, and then transferred the prepared flexible printed circuit board (FPCB) to the VB-EP material. Once the FPCB was transferred to the VB-EP substrate, we initiated the creation of multilayer connections. Given that the proposed BWCEH system needs to be adapted to various non-flat surfaces and faces contact force, the reliability of conductive vias is extremely crucial. We first employed a 200 µm thick copper wire to connect the upper layer to the ground plane through low-temperature welding, and then infused silver paste into the vias, ensuring reliable multilayer interconnections. After completion of the system integration and multilayer interconnection, we encapsulated the system in a transparent polystyrene (PS) mold. A layer of PDMS-based epoxy resin solution was poured into the mold as the bottom of the self-healing encapsulation. Once it nearly solidified, we placed the system in the mold and poured a thin layer of epoxy solution onto the system surface. After 24 h of heating (60°C), the system could then be removed from the mold, and it had been fully covered by the SH-EP elastomer. Compared with the commercial tapes and encapsulations, the self-healable SH-EP encapsulation exhibits obvious advantages in terms of interfacial adaptability and long-term reliability.

The fabricated system is shown in Fig. 3b. Due to the unique transformative ability, the fabricated system can arbitrarily transform its shape and adapt to various uneven surfaces after heating, and maintain its shape after cooling while regaining excellent mechanical strength, which is demonstrated in Video S1. In the soft state, the VB-EP substrate will possess a relatively similar Young's moduli (11.2 MPa) with the SH-EP encapsulation (1.6 MPa), thereby avoiding interfacial delamination or structure damage/failure due to mechanical stress concentration. Meanwhile, in the rigid state, the overall mechanical performance of the system is determined by the VB-EP substrate. In Fig. 3c, we placed a 1.5 kg load on the BWCEH system, and the load had no impact on the system's profile. Experimental results demonstrate that our system exhibits superior shape retention ability and mechanical strength at room temperature, which are crucial for protecting microwave devices and supporting stable electromagnetic performance. We dropped the system from a maximum height of 2 meters, and the system remained undamaged after suffering strong contact force. Furthermore, we intentionally pierced the self-healable encapsulation with a sharp blade, and exposed the system to the air at room temperature for 24 h. Fig. 3d illustrates the damage experiment of the self-healable encapsulation, revealing that the encapsulation has fully healed except for the surface scars.

Except for contact force or damage, the bio-inspired design strategy can protect the system from liquid corrosion during prolonged operation. We investigated the antiwetting behavior of the skin-like encapsulation. As shown in Fig. 3e, the water contact angle of the SH-EP film is measured to be 102.6°, indicating good hydrophobicity. After being immersed in water for 15 d, the shape and mechanical properties of SH-EP polymer can be stabilized. Considering practical applications in harsh environments, we further tested the chemical stability of the prepared encapsulants. We immersed the SH-EP films in strong acidic (1 M HCl, pH = 1), strong alkaline (1 M NaOH, pH = 14) and neutral solutions (deionized water, pH = 7) at ambient temperature for 48 h (Fig. S14); the treated films still maintained their mechanical properties comparable to the original ones (Fig. 3f). The FT-IR tests also show no significant structural changes in the treated films, indicating their reliability for long-term applications (Fig. 3g). In addition, we also explored the stability of VB-EP substrate material in high-humidity environments. After being immersed in water for 15 d, the electromagnetic characteristics of the VB-EP material remained stable, which is shown is Fig. S15. These experimental results demonstrate the bio-inspired system's exceptional ability to resist contact force, mechanical damage, and liquid exposure.

### Modulate ambient electromagnetic waves for ultra-low-power communication

A major obstacle hindering the large-scale expansion of the IoT network is the excessively complex power delivery deployment, leading to the enormous cost of infrastructure construction. At present, the vast majority of IoT terminals rely on active communication, implying that the terminal needs to generate massive electromagnetic waves to achieve data exchange. Taking the commercial Bluetooth 4.0 module as an example, in Level 1 operation mode, about 100 mW of energy is required to generate electromagnetic waves. Currently, the power consumption of microprocessors and sensors can be reduced to microwatt-level. It is evident that, for current IoT terminals, the power consumption of the wireless communication has become overwhelmingly dominant.

**Figure 4.**
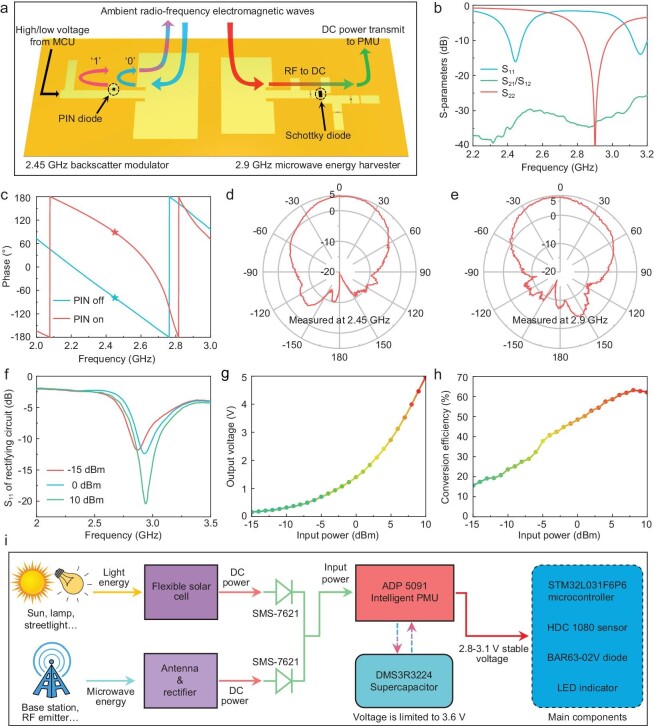
Backscatter modulator and hybrid energy harvesting system. (a) Operational illustration of the backscatter modulator and the RF energy harvester. MCU: microcontroller unit. (b) S-parameters of the 2.45 GHz antenna and 2.9 GHz antenna. S_11_ represents the impedance matching of the 2.45 GHz patch antenna, while S_22_ represents the 2.9 GHz patch antenna. The S_21_/_12_ represents the isolation between the 2.45 GHz antenna and 2.9 GHz antenna. (c) Reflection phase difference of the backscatter circuit. (d) Radiation pattern of the 2.45 GHz patch antenna; the maximum realized gain is 4.91 dBi. (e) Radiation pattern of the 2.9 GHz patch antenna; the maximum realized gain reaches 6.68 dBi. (f) Impedance matching properties of the rectifying circuit. The output voltage (g) and conversion efficiency (h) of the rectifying circuit. To estimate the conversion efficiency, the circuit is connected with a 4 kΩ resistor load. (i) Illustration of the hybrid energy harvesting and management system.

Therefore, to achieve battery-free IoT nodes, it is imperative to significantly reduce the power consumption caused by the wireless communication process. Thus, we employ the backscatter communication scheme to achieve wireless data transmissions. Backscatter communication allows devices to modulate external electromagnetic waves to realize wireless information transmission, eliminating the need for generating microwave energy [45]. We created a 2.45 GHz backscatter circuit and an efficient patch antenna on the VB-EP substrate, constituting an ultra-low-power backscatter modulator, which is depicted in Fig. 4a–b, and detailed geometric parameters are shown in Figs S16–S17. When a bit of data is ‘1’, the microcontroller's control pin outputs a high-level voltage to activate the PIN diode. When the temperature data bit is ‘0’, the microcontroller's control pin switches to the low level, and the PIN diode will switch to the cut-off state. While the microcontroller controls the PIN diode's states, the backscatter circuit will produce ∼160-degree phase difference, which is shown in Fig. 4c. Therefore, the external base station can demodulate the phase of the reflected electromagnetic waves to achieve a Binary Phase Shift Keying (BPSK) wireless communication. Thanks to the low insertion loss of the backscatter circuit (<1.6 dB, Fig. S18) and the high radiation efficiency (realized gain reaches 4.91 dBi, Fig. 4d) of the patch antenna, the fabricated backscatter modulator exhibits outstanding wireless communication quality. Meanwhile, the transformative microwave structure maintains stable electromagnetic performance when it is bent (Fig. S19). Unlike conventional flexible devices, the transformable backscatter communication modulator exhibits superior structural stability to support reliable electromagnetic signal modulation even under contact force, which is crucial for the stability of wireless communication.

To further reduce the power consumption, we optimize the clock frequency of the microcontroller and disable all unnecessary built-in functions. We program the microcontroller to send one data packet every second, and thus most of the time the microcontroller operates in the ultra-low-power standby mode. By introducing the backscatter communication structures and optimizing the operation logic of the microcontroller, the total power consumption of our BWCEH system is less than 5 mW, which is much smaller than that of the current IoT systems based on active communications. Meanwhile, we simulated the specific absorption rate (SAR) of the backscatter wireless communication system after it had been installed on the human body. The electromagnetic model and simulated results are shown in Fig. S20. It can be observed that the simulated SAR value of the backscatter communication is far below the standard limits, ensuring safety for wearable applications.

### Microwave-solar hybrid energy harvesting and management

The backscatter communication scheme significantly reduces the power consumption of wireless communications. On this basis, the introduction of an efficient energy harvesting and management system facilitates a battery-free wireless communication terminal [46]. Solar energy, being the most common and abundant energy source, is widely used in IoT fields. However, the output power of the solar cells is extremely sensitive to incident light. Therefore, in indoor or rainy conditions, the output power of the solar energy will decrease to the micro-watt level, which is insufficient to meet the needs of wireless communication terminals. To address this, fusing other types of energy harvesting methods like triboelectric nanogenerator (TENG) [47], thermoelectric effects [48] and microwave energy transmission [49] with photovoltaics is a promising solution to achieving battery-free distributed systems with better environmental adaptability. In this work, we fuse the flexible solar cell with an efficient microwave energy harvester to power the BWCEH system, and design an energy management system to further enhance the operational stability.

The schematic diagram and detailed geometric parameters of the microwave energy harvester are depicted in Fig. 4a and Figs S21–S22. The microwave energy harvester comprises a patch antenna and an efficient rectifying circuit operating at 2.9 GHz (the S_11_ of the antenna is shown in Fig. 4b). The antenna captures incident electromagnetic waves, while the rectifying circuit converts the microwave energy into DC power. The measured gain of the 2.9 GHz transformative antenna reaches 6.68 dBi, ensuring that most of the incident energy can be received by the rectifying circuit (Fig. 4e). The impedance matching characteristics, output voltage, and conversion efficiency of the rectifying circuit are illustrated in Fig. 4f–h, demonstrating good matching characteristics and excellent conversion efficiency. The maximum conversion efficiency of the rectifying circuit reaches 63%, and the rectifying circuit exhibits reliable performance across a wide range of input RF power levels. In addition, the antenna and rectifying circuit can maintain consistent efficiency under structural deformation (Figs S23–S25). These results demonstrate that the 2.9 GHz rectenna possesses excellent electromagnetic performance and can effectively accommodate ambient electromagnetic energy harvesting and wireless microwave power transfer (MPT).

The power strategy of the hybrid energy harvesting and management is illustrated in Fig. 4i. A flexible film solar cell with a maximum output power of 500 mW is employed in this study. As the energy levels of microwaves and solar energy are unlikely to be in the same order of magnitude, we employ an anti-backflow protection circuit to safely integrate the two energy sources together. After energy merging, they are fed into an ADP5091 intelligent power management unit (PMU). This PMU can automatically store the collected energy in a supercapacitor, and deliver a stable power supply to the microwave system. When the supercapacitor voltage exceeds 2.7 V, the PMU initiates the supply of electric power to the BWCEH system. As the voltage of the supercapacitor continues to rise, the PMU's output voltage is maintained at ∼3.1 V to ensure reliable operation of the electronic components. Simultaneously, the PMU will continuously monitor the voltage of the supercapacitor for safety. When the voltage reaches 3.6 V, the PMU will no longer replenish energy to the supercapacitor.

### All-scenario, battery-free wireless sensing and communication

Benefiting from its transformative ability, excellent reliability, ultra-low power consumption, efficient energy harvesting and management, and self-healable elastomeric encapsulation, the proposed BWCEH system enables battery-free wireless sensing and communication in nearly all environments, making it highly promising for constructing a passive and maintenance-free IoT network. To validate our strategy, we integrated a digital high-precision temperature sensor with the BWCEH system, and performed comprehensive measurements to estimate the system's wireless communication quality, operation stability, and environmental adaptability.

**Figure 5.**
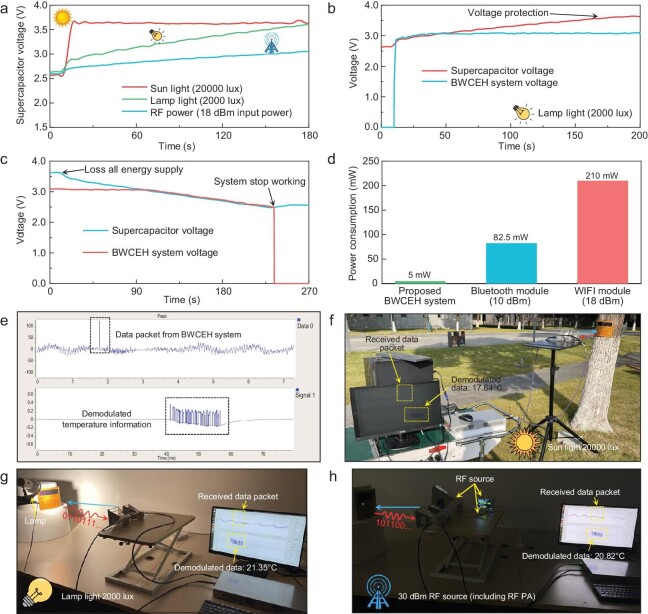
Battery-free wireless communication and sensing of the BWCEH system. (a) Charging curves of the supercapacitor in different environments. (b) Measured voltage of supercapacitor and system under 2000 lux lamp light. (c) Voltage status of the BWCEH system when it loses all electromagnetic energy supply. (d) Power consumption comparison between the BWCEH system and other conventional communication modules. The power consumption of Bluetooth and Wi-Fi were estimated by an Ai-Thinker PB-02 Bluetooth module and an ATK-ESP8266 Wi-Fi module. (e) Temperature data packets and demodulated information received by the USRP system. Wireless communication experiments under (f) sunlight, (g) indoor lamp light, and (h) RF-driven conditions.

The system voltage curve can reveal the relationship between the operation status and environments, which are illustrated in Fig. 5a–c. As shown in Fig. 5a, the bio-inspired system obtains abundant energy supplies under outdoor sunlight, and the supercapacitor is fully charged in 9 s. Indoors, under a desk lamp of 2000 lux, it takes ∼3 min for the system to fully charge the supercapacitor. Microwave energy is much weaker than solar energy, and thus the system requires a longer time to replenish electronic power under the RF-driven condition. Despite the considerable difference in energy densities across different environments, owing to the ultra-low-power design, once the PMU is activated, the BWCEH system can immediately come into stable operation when the PMU starts supplying voltage, which is depicted in Fig. 5b. Furthermore, as shown in Fig. 5c, the ultra-low-power design allows the system to maintain stable operation for nearly 4 min even after losing all electromagnetic energy sources. Fig. 5d presents a comparison of power consumption between the BWCEH system and other conventional communication modules. Compared to a WI-FI module, our system's total power consumption is only ∼2.38% of the former's communication power consumption.

To evaluate the wireless communication performance, we employed a universal software radio peripheral (USRP) system to simulate a base station and demodulate the electromagnetic waves reflected by the BWCEH system. We defined an M-sequence authentication mechanism in the BWCEH system and USRP system to further enhance the accuracy of the wireless data transmission. Fig. 5e shows the temperature data packets received by the USRP system and the demodulated digital information. We set up a wireless communication measurement platform in outdoor sunlight, indoor lighting, and dark environments to evaluate the communication performance of the system (Fig. 5f–h, Video S2–S4). Experimental results indicate that the BWCEH system can support stable wireless sensing and communication under all these conditions. When the RF output power of the USRP system is 5 dBm and there is sufficient light, the system can achieve stable sensing data transmission within a range of 3 m. In completely dark environments, we employed an RF source, a power amplifier, and a horn antenna to form a 30 dBm microwave energy emitter. When the microwave transmission distance is 50 cm, the communication quality of the system can be comparable to that in light-driven mode.

**Figure 6.**
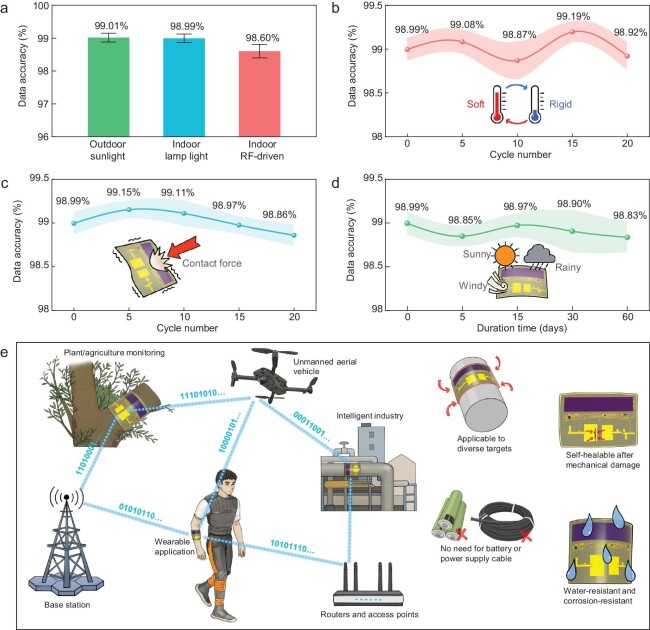
A systematic study on environmental adaptability and long-term stability of the BWCEH system. (a) Communication performance in different operational scenarios. (b) Wireless transmission accuracy after repeated heating, bending and cooling. (c) Wireless transmission accuracy after withstanding contact forces. (d) Long-term stability of the system in natural environments. (e) Future passive and maintenance-free IoT networks based on the BWCEH system.

To estimate environmental adaptability and long-period durability, we conducted comprehensive measurements of communication quality under various conditions. By comparing received temperature data with high-precision thermometers, we evaluated the accuracy of wireless transmission, thereby revealing the system's wireless communication performance. Fig. 6a illustrates the system's adaptability to different operational scenarios. Under outdoor sunlight and indoor lighting, the average data accuracy of the backscatter communication can exceed 98.99%. Even in complete darkness, the system still supports high-quality backscatter communication through microwave power transmission. Furthermore, the system can maintain consistent wireless performance under varying degrees of bending deformation, which is shown in Fig. S26. In addition, we also explored the impact of polarization mismatching on wireless communication quality of the BWCEH system. Experimental results prove that, even if the BWCEH is worn on the human body, it will maintain stable wireless communication under polarization mismatching conditions (Fig. S27). These experiments demonstrate that the BWCEH system exhibits reliable wireless communication performance under conditions of structural deformation, polarization mismatching, and in various operational environments.

We then performed multiple cycles of ‘heating-bending-cooling’ tests on the bio-inspired system to examine its electromagnetic stability. As shown in Fig. 6b, the frequent phase transition and structural bending of the system have negligible influences on its electromagnetic performance. After 20 cycles of testing, the data accuracy remains >98.87%, confirming the exceptional structural stability of the BWCEH system. Next, cyclic loading tests are conducted to verify the bio-inspired system's tolerance to contact forces. We randomly dropped the system from a height of 1 m and tested its communication performance after withstanding high contact forces. After suffering repeated violent impacts, the average transmission accuracy remained between 98.86% and 99.15% (Fig. 6c). We exposed the BWCEH system to natural environments for a 2-mo durability test, and the measured communication quality is presented in Fig. 6d. Despite experiencing diverse weather conditions, the system held stable electromagnetic performance. In addition, we explored the wireless communication ability of the BWCEH system in high-humidity environments. Experimental results indicate that even in high-humidity (>90%) environments, the average data accuracy of the backscatter wireless communication can still reach 98.88%, demonstrating stable and reliable wireless communication quality (Fig. S28). These features allow the BWCEH system to basically eliminate the need for frequent post-maintenance, thereby significantly diminishing the long-period operation costs of distributed IoT networks.

Experimental results verify the performance of the BWCEH system in wireless communication, environmental adaptability, transformative ability and long-period reliability. Due to equipment limitations, the RF output power of our USRP system can reach merely 10 dBm. However, communication nodes in daily life, such as wireless routers and small base stations, can achieve a microwave output power of 33–43 dBm. This implies that if connected to these communication nodes, the wireless communication distance of the BWCEH system can extend to tens of meters or even longer. Meanwhile, in terms of microwave power transfer, the proposed system also has the potential to integrate with existing high-power base stations (e.g. B41, n77 and n78 band) through spectrum expansion. In the future, combining reconfigurable antenna array technology, unmanned aerial vehicles, and reconfigurable metasurface techniques [50,51], a single communication node or base station may wirelessly monitor and power all distributed BWCEH systems within the range of tens of meters, which is depicted in Fig. 6e. Compared with previous TESs, the proposed bio-inspired BWCEH system demonstrates outstanding electromagnetic performance and does not need to rely on any replaceable batteries or power supply cables, showcasing advantages in terms of installation complexity, maintenance costs, and long-term sustainability. Moreover, the skin-like self-healable encapsulation endows our system with superior durability, and protects the system from contact force, chemical corrosion and high-humidity environments. These unique advantages of the BWCEH system overcome the challenges of unstable electromagnetic performance, difficult power supply deployment, and high maintenance costs faced by current IoT network expansion. At the same time, the proposed system enables various interfaces to be compatible with different types of sensors and other smart devices. In the future, the proposed BWCEH system will serve as the foundational nodes to construct a vast, seamless, passive IoT network, connecting humans, smart devices, and the physical world.

## CONCLUSION

In this work, we report a bio-inspired microwave system to modulate and harvest ambient electromagnetic waves for constituting future passive IoT networks. Inspired by the body structure of vertebrates, we propose a strategy for creating high-performance microwave systems with transformative ability, outstanding reliability, battery-free operation, and self-healable characteristics. Leveraging this biomimetic approach, our system combines excellent flexibility with outstanding robustness, allowing it to adapt to diverse objects while maintaining stable electromagnetic performance and superior reliability. The skin-like soft encapsulation exhibits exceptional strength, remarkable chemical stability and room-temperature self-healing ability, providing durable protection against contact forces and corrosion. Furthermore, the fusion of an ultra-low-power backscatter communication scheme and solar-microwave hybrid energy harvesting technique enables stable, long-range wireless sensing and communication without the need for replaceable batteries or power supply cables. The multidisciplinary innovation in design strategy, material science, microwave engineering, and wireless communication effectively overcomes the critical challenges in IoT network expansion. We believe the proposed BWCEH system holds immense potential in various applications, such as wearable devices, agricultural monitoring, wireless communications, and smart cities.

## MATERIALS AND METHODS

*Materials and general measurement*: Liquid thiol-terminated polysulfide oligomer (LP3, Mn = 1000) was supplied by Thiokol Co., Ltd, Japan. (Epoxypropoxypropyl)dimethoxysilyl terminated polydimethylsiloxane (PDMS-epoxy, 80–120 cSt.) was purchased from Gelest. Tris(dimethylaminomethyl)phenol (DMP30, 80%) was purchased from Shanghai Bide Pharmaceutical Technology Co., Ltd. Epoxy resins based on bisphenol A diglycidyl ether (GELR-128), with an epoxide equivalent of 182–192 g eq^−1^ was purchased from Macklin. Poly(propylene glycol) bis(2-aminopropyl ether) (D230) and 4-aminophenyl disulfide were obtained from Aladdin Reagent Co. All commercially obtained chemicals were used as received without further purification.

Thermogravimetric analysis (TGA) was carried out on a simultaneous SDT 2960 thermal analysis system from 30°C to 600°C with a heating rate of 10°C min^−1^ under N_2_ atmosphere. Differential scanning calorimetry (DSC) experiments were performed using a Mettler-Toledo DSC1 STARe analyzer under dry N_2_ atmosphere (50 mL min^−1^) from −60°C to 80°C at the speed of 10°C min^−1^. The rheological behaviors were evaluated on a TA Instruments DHR-2 system. Temperature sweeps were run from 0°C to 120°C at a rate of 2°C min^−1^ and a frequency of 1 Hz with 20 mm parallel plates. Contact with the sample was maintained by the auto-compression feature set to 1 ± 0.1 N. FT-IR spectra were recorded on a Bruker Tensor 27 FTIR spectrophotometer ranging from 4000 to 400 cm^−1^.

*Synthesis of SH-EP films and VB-EP films*: LP3 (4.0 g), PDMS-epoxy (2.0 g) and 4-aminophenyl disulfide (0.1242 g), together with DMP-30 (0.09 g) as the catalyst were thoroughly mixed. After removing bubbles with vacuum, the epoxy resins were poured into a silicon mold and placed in an oven at 60°C for 12 h to completely cure. A yellow transparent film was obtained after peeling off from the silicon mold. As for VP-EP films, the preparation steps were similar to those for SH-EP films, except that the masses of D230 and GELR-128 were 21.3 g and 48.0 g, respectively.

*Wireless sensing and communication measurements of the BWCEH system*: Wireless sensing measurements were conducted by the Luowave N310-LW USRP system. The communication scheme of the USRP system was set to BPSK, and the output power was set to 5 dBm. We used a pair of horn antennas and connected them to the USRP system to act as the transmitting and receiving antennas. The USRP system generated carrier waves, and demodulated waves reflected by our BWCEH system. Data packets of the temperature data and demodulated digital signals can be directly observed on the user interface. The recovered data were stored in a TXT file by the USRP system. In the outdoor and indoor conditions, we use a AS803 digital lux meter to estimate the intensity of the incident light. In the RF-driven condition measurement, we integrated a SG-3000-PRO power source, an RF amplifier and a high-gain horn antenna to form an RF power emitter, and wirelessly transmitted energy to the system. The voltage status of the system was monitored by a Keysight DSOX3024T digital storage oscilloscope.

More detailed information of the experimental method and fabrication process is illustrated in the Supplementary material.

## Supplementary Material

nwae435_Supplemental_Files
