# Supplementary material for: A bio-inspired microwave wireless system for constituting passive and maintenance-free IoT networks
Source: Natl Sci Rev. 2024 Dec 9;12(2):nwae435. doi: 10.1093/nsr/nwae435 (PMC11737395; doi:10.1093/nsr/nwae435)
Supplement: nwae435_Supplemental_Files [file nwae435_supplemental_files.zip › Supplementary Information.pdf]

Supplemental information

A bio-inspired microwave wireless system for constituting passive and maintenance-free IoT networks

Buyun Yu[1,3,#], *Hong-Qin Wang*[2,#], *Lu Ju*[1,3], *Ke-Xin Hou*[2], *Zhi-Da Xiao*[1,3], *Jun-Lin Zhan*[1,3], *Chao Zhang*[1,3], *Hao Chen*[1,3], *Binghao Wang*[4], *Zhen-Guo Liu*[1,3], *Ying-Shi Guan*[5], *Cheng-Hui Li*[2,*], *Tie Jun Cui*[1*], *Wei-Bing Lu*[1,3,*]

[1]State Key Laboratory of Millimeter Waves, School of Information Science and Engineering, Southeast University, Nanjing 210096, China

[2]State Key Laboratory of Coordination Chemistry, School of Chemistry and Chemical Engineering, Collaborative Innovation Center of Advanced Microstructures, Nanjing University, Nanjing 210023, China

[3]Center for Flexible RF Technology, Frontiers Science Center for Mobile Information Communication and Security, Southeast University, Nanjing 210096, China

[4]School of Electronic Science and Engineering, Southeast University, Nanjing 210096, China.

[5]School of Chemistry and Chemical Engineering, Southeast University, Nanjing 210096, China.

[#]These authors contributed equally to this work.

*Correspondence to: wblu@seu.edu.cn; tjcui@seu.edu.cn; chli@nju.edu.cn.

Additional information of the experimental methods and fabrication process

Dielectric characteristic measurement of the VB-EP microwave substrate: The dielectric characteristics of the VB-EP microwave substrate was obtained by a split post dielectric resonator (QWED F-SPDR-5.1). The split post dielectric resonator has an original resonance at the frequency of 5.1 GHz. When a 1 mm thick VB-EP substrate is inserted into the split post dielectric resonator, the resonant frequency and Q factor will shift to a lower frequency. With the help of the software provided by QWED, we can obtain the dielectric constant of the VB-EP material by evaluating the frequency shift. By evaluating the variation of Q factor, we can calculate the dielectric loss of the VB-EP material.

Fabrication the BWCEH system: The 100 μm polyimide FPCB was manufactured by Shenzhen JLC group company. Most of the components were integrated on the FPCB by SMT process. Schematic diagrams and the board layouts of our BWCEH system were designed using Altium Designer (20.2.2), and the detailed schematic diagrams are shown in Fig. S29. The components include 0603 footprint passive elements (resistors, capacitors and inductors), STM32L031F6P6 microcontroller, HDC1080DMBR temperature sensor, ADP5091-2 power management unit, DMS3R3224RS supercapacitor, series-pair SMS7630-005LF Schottky diode, Infineon BAR63-02V silicon PIN diode, and green LED. The PIN diode, Schottky diode, RF capacitors and RF inductors were welded on the FPCB manually. The VB-EP substrate was drilled by 6040B laser machine from Han's Yueming Laser Company. For the integration of the BWCEH system, we spin-coated the Mechanic 480 adhesive on the VB-EP substrate by SETCAS KW-4L spin coater. JY50 nano-silver paste was utilized to form the metallic ground plane and conductive vias.

Electromagnetic simulations: Full-wave simulation was conducted for electromagnetic simulation to study the impedance matching properties, radiation performance and electromagnetic field distributions of the two antennas. These simulations were performed in CST 2022 microwave studio. The backscatter circuit and rectifying circuit were simulated in PathWave Advanced Design System 2020. The SPICE model of the BAR63-02V diode and SMS-7630-005LF were obtained in datasheets from Infineon and Skyworks.

Electromagnetic measurements of antennas, backscatter circuit and rectifying circuit: The S-parameters measurements of the antennas, backscatter circuit and rectifying circuit were performed by our vector network analyzers (model: Rohde&Schwarz ZNB40, Keysight N5227B and Transcom T5260C). The RF-to-DC conversion efficiency measurements of the rectifying circuit were performed by a SG-3000-PRO RF power source. In the bend-state measurements, these microwave devices were conformed to cylindrical foams (diameter is 15 cm) and connected to the vector network analyzer to estimate RF performance stability. The realized gain of the antenna was obtained in an anechoic chamber in Southeast University (Fig. S30).

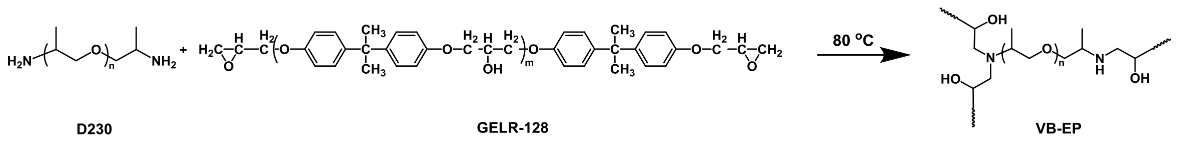

Fig. S1. Synthesis route of VB-EP.

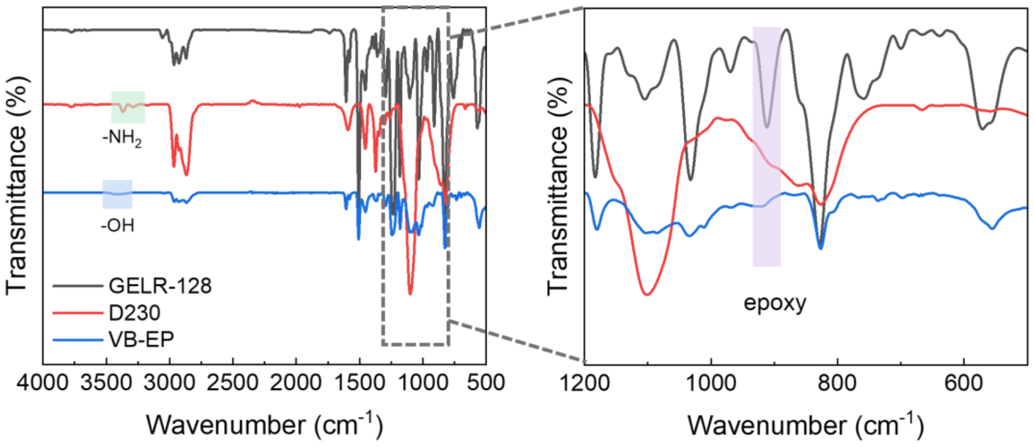

Fig. S2. FTIR spectra of reactants and the product VB-EP. The characteristic peaks of $-NH_2$ (3364 cm^{-1}, 3284 cm^{-1}) from D230 and the epoxy group (910 cm^{-1}) of GELR-128 disappeared, while a new characteristic peak of -OH (3411 cm^{-1}) appeared in the product VB-EP.

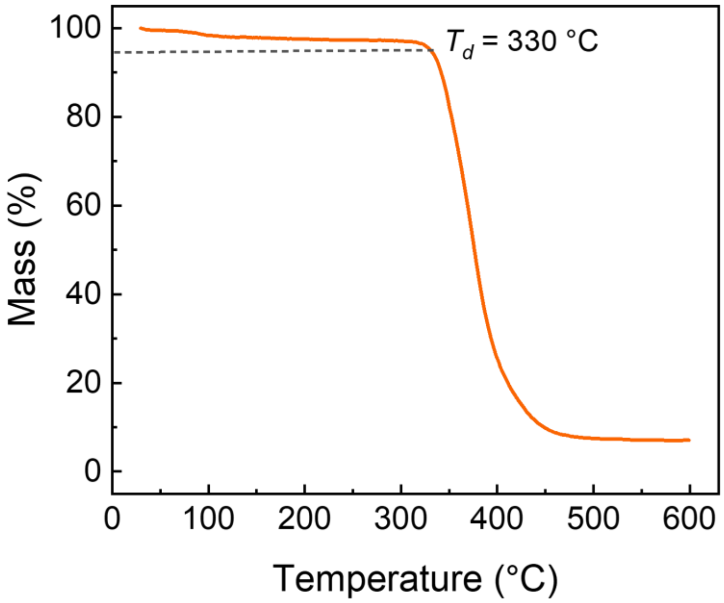

Fig. S3. TGA curve of VB-EP.

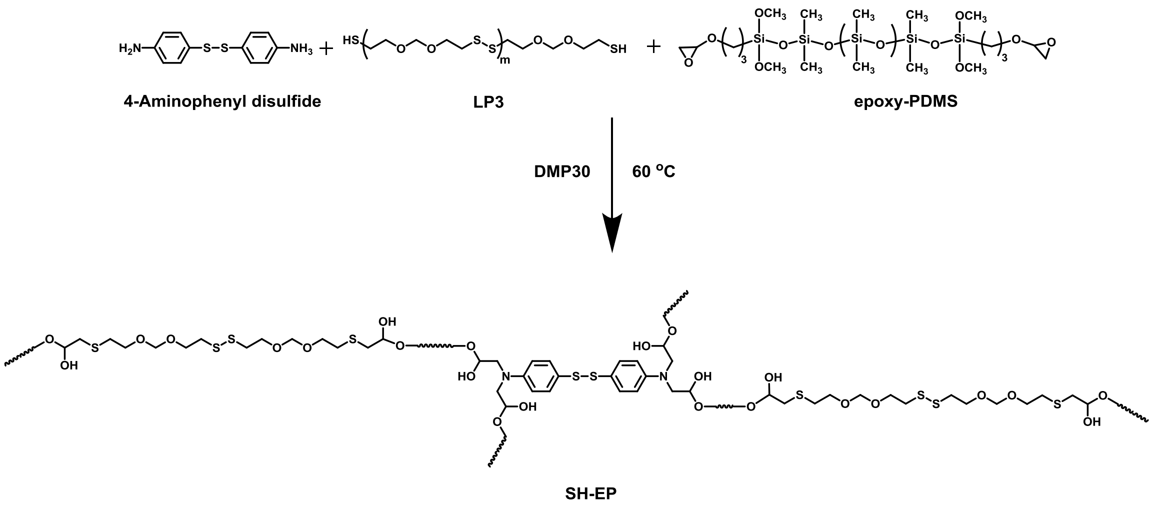

Fig. S4. Synthesis route of SH-EP.

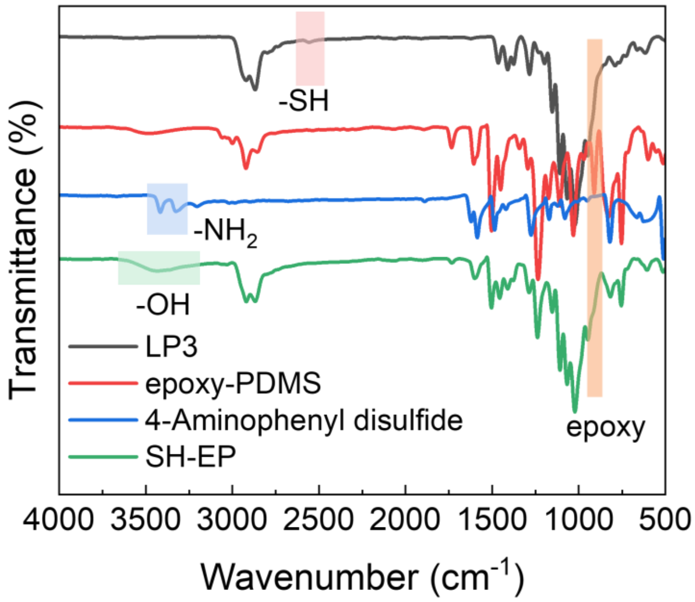

Fig. S5. FTIR spectra of reactants and the product SH-EP. The characteristic peaks of -NH_2 (3419 cm^{-1}, 3326 cm^{-1}) from 4-Aminophenyl disulfide, the thiol group (2554 cm^{-1}) from LP3 and the epoxy group (910 cm^{-1}) of epoxy-PDMS disappeared, while a new characteristic peak of -OH (3441 cm^{-1}) appeared in the product SH-EP.

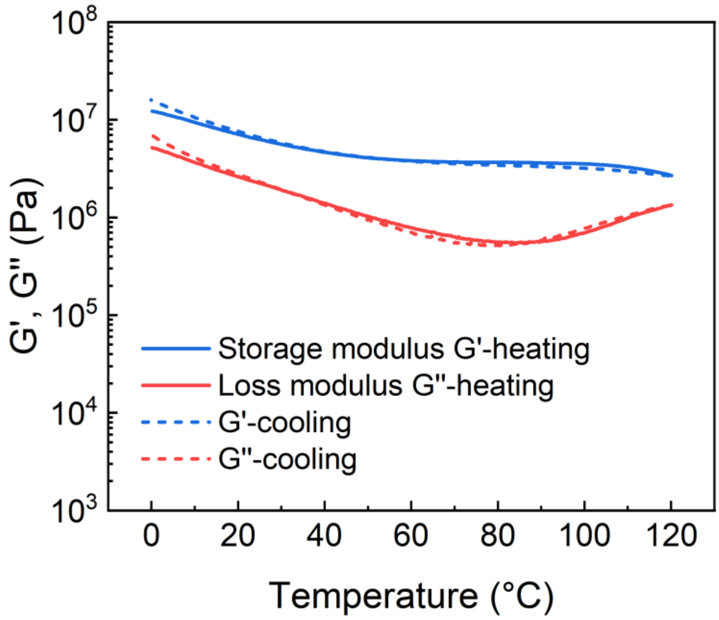

Fig. S6. Dynamic oscillatory temperature sweeps of SH-EP.

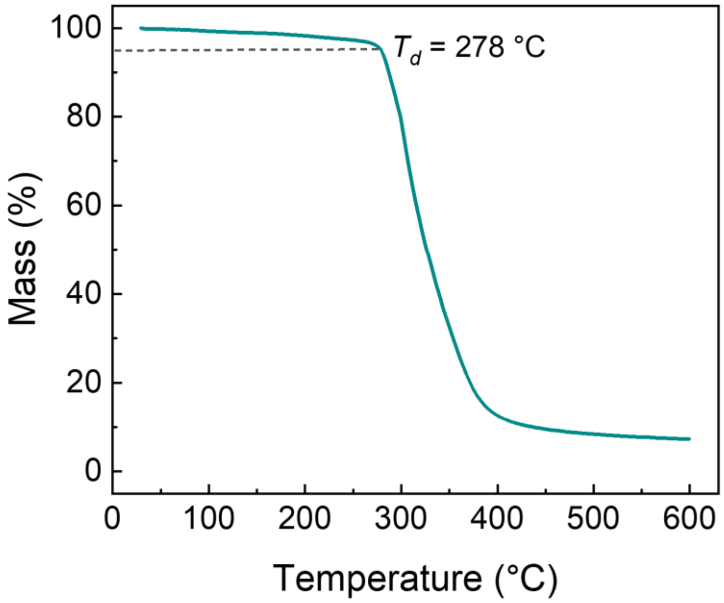

Fig. S7. TGA curve of SH-EP.

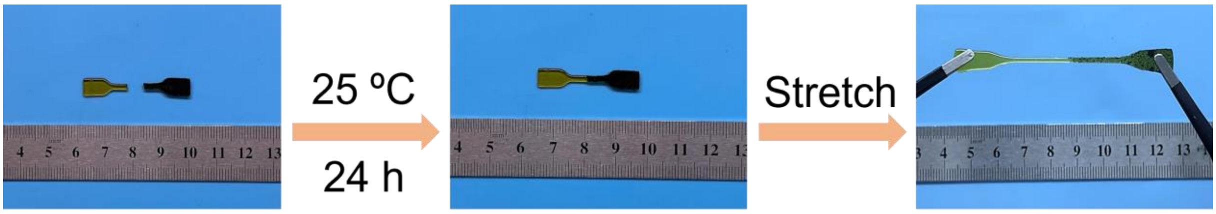

Fig. S8. Photographs of self-healing between either two freshly cut surfaces (yellow and blue) of SH-EP samples at 25 ℃ for 24 h.

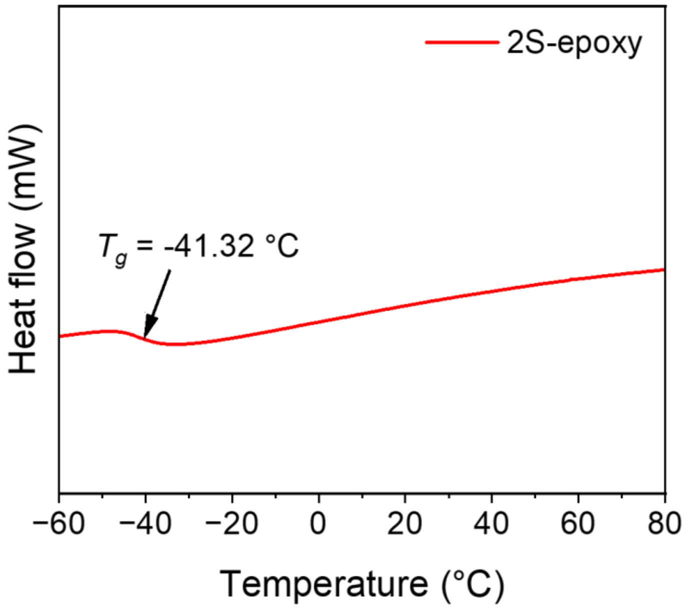

Fig. S9. DSC curve and the glass transition temperature (T_g) of SH-EP.

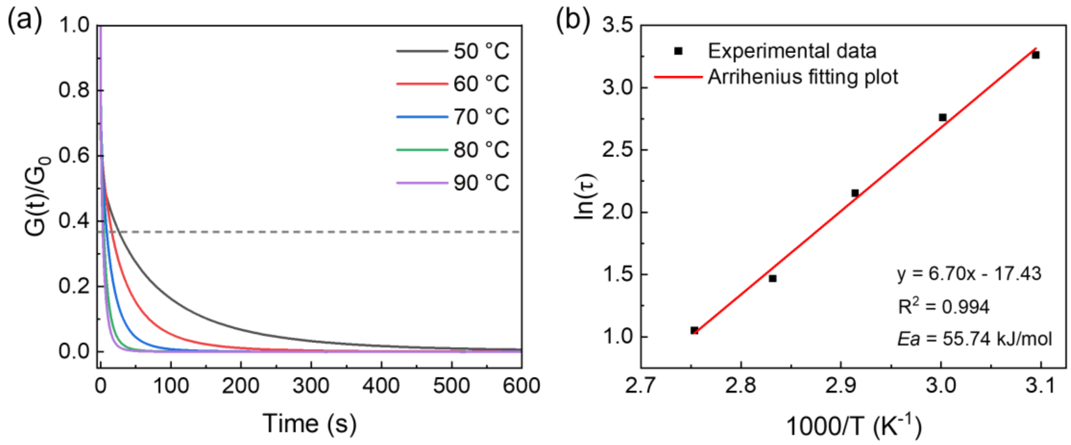

Fig. S10. Dynamic mechanical behavior of the SH-EP film. (a) Normalized stress relaxation curves of the SH-EP film. The measurements were performed at a shear strain of 2% at different temperatures from 50 ℃ to 90 ℃. (b) Arrhenius fitting plots of the SH-EP film.

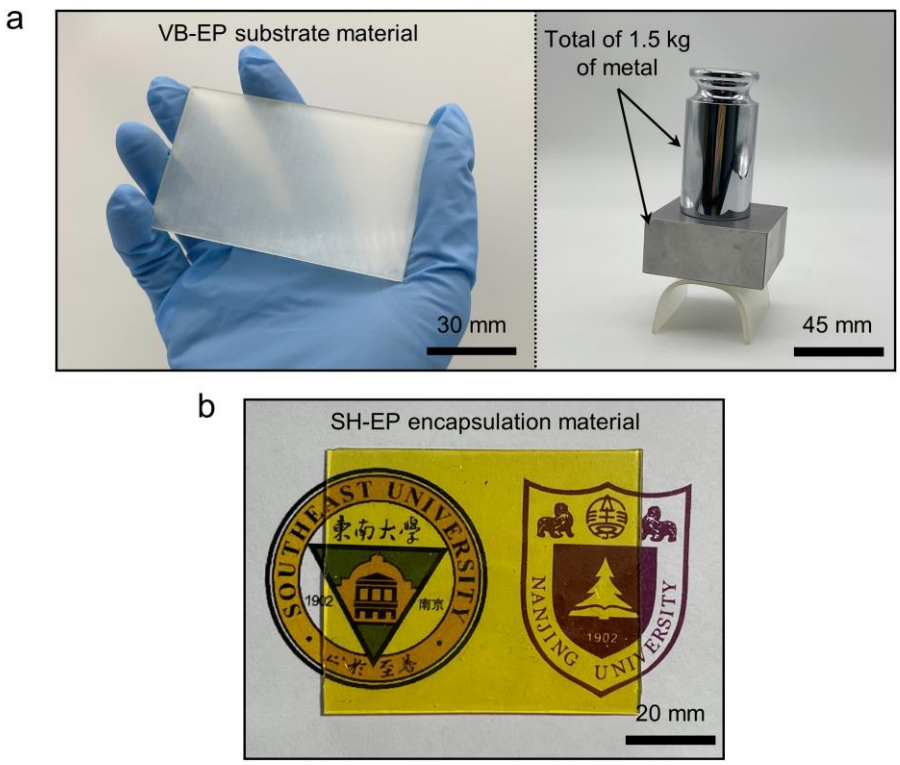

Fig. S11. (a) Photograph of the VB-EP substrate, and demonstration of the mechanical strength and shape retention ability. (b) Photograph of the prepared SH-EP films.

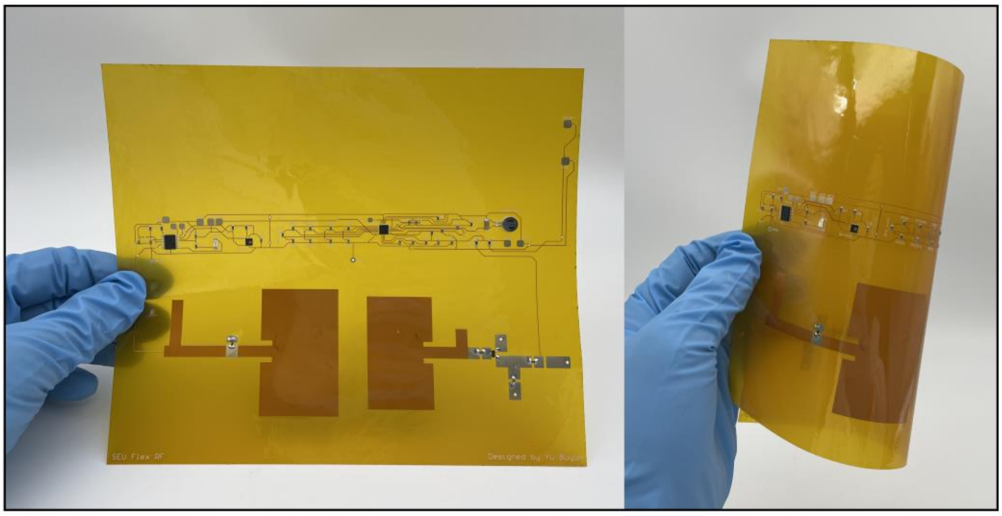

Fig. S12. Images of the fabricated Polyimide-based FPCB. Most of the components were integrated on the FPCB by SMT process. The PIN diode, Schottky diode, RF capacitors and RF inductors were welded on the FPCB manually.

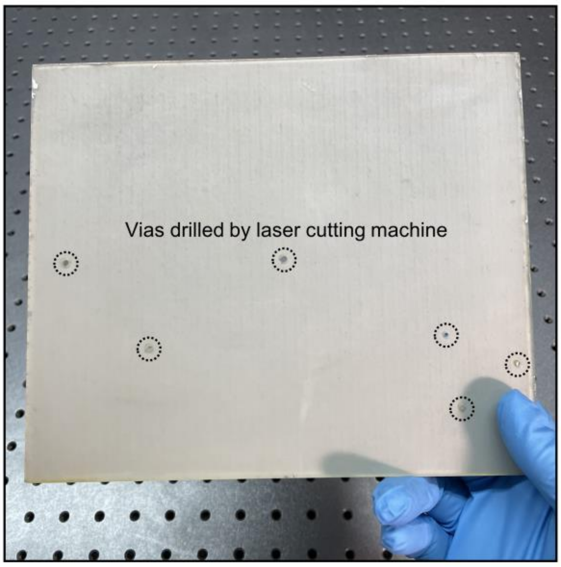

Fig. S13. Image of the drilled VB-EP substrate.

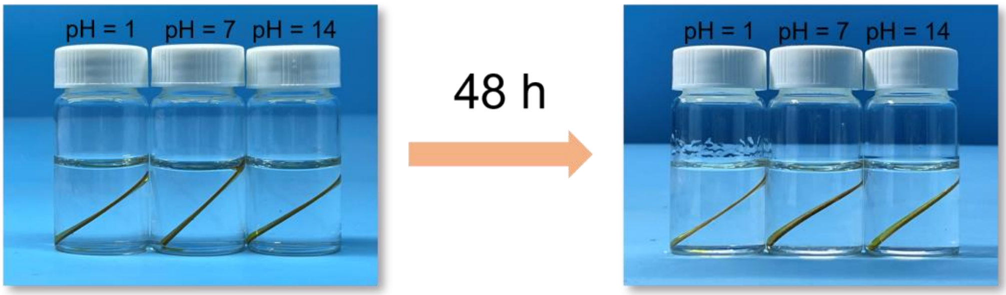

Fig. S14. Images of the SH-EP films immersed in strong acidic (1M HCl, pH = 1), strong alkaline (1M NaOH, pH = 14) and neutral solutions (deionized water, pH = 7) at ambient temperature for 48 h.

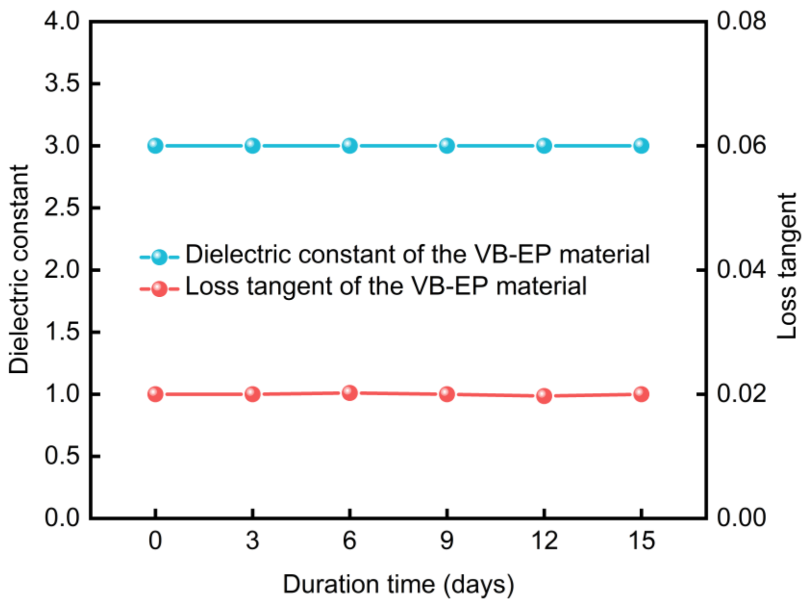

Fig. S15. The electromagnetic characteristics of the VB-EP material after being immersed in water for 15 days at room temperature.

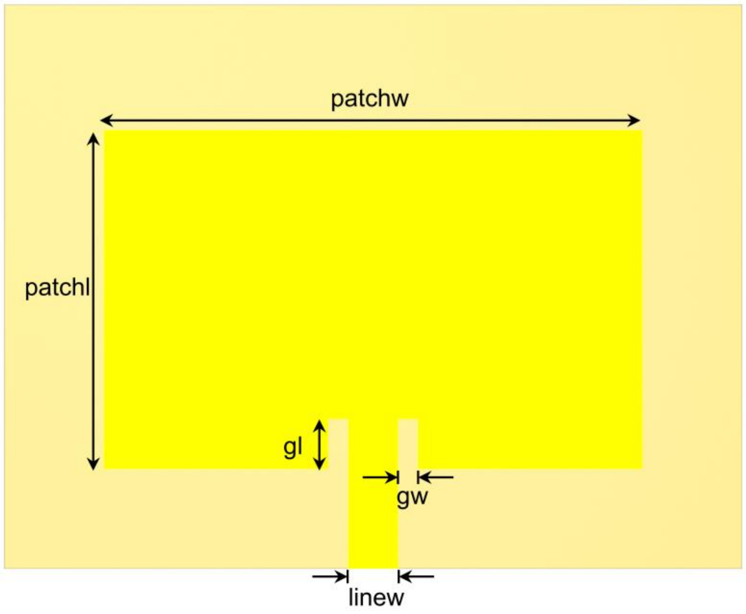

Fig. S16. Detailed geometric parameters of the 2.45 GHz transformative patch antenna. patchw = 54 mm, patchl = 34 mm, gw = 2 mm, gl = 5 mm, linew = 5 mm. The thickness of the VB-EP substrate is 2 mm, and the bottom of the antenna is cover by a layer of printed silver film.

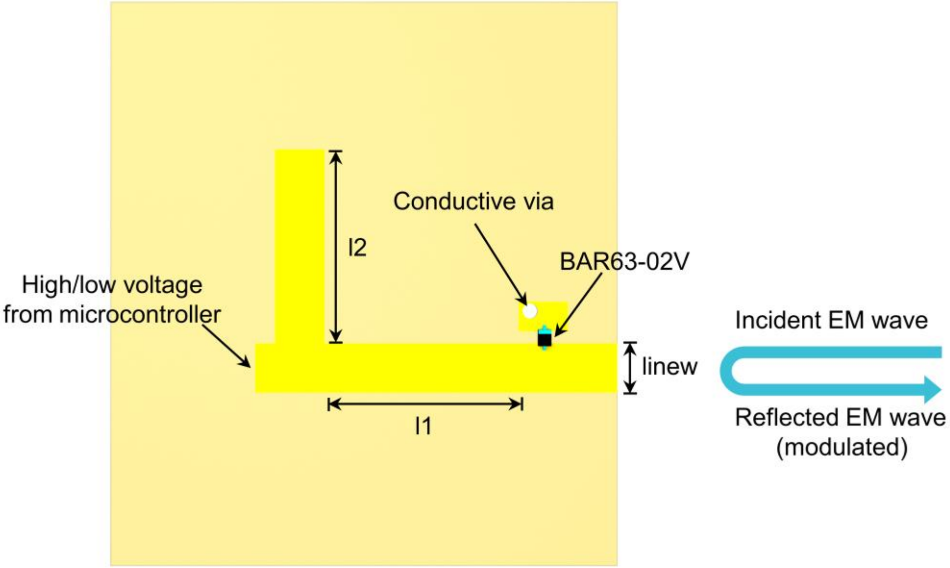

Fig. S17. Detailed geometric parameters of the 2.45 GHz transformative backscatter circuit. l1 = 19.6 mm, l2 = 19.6 mm, linew = 5 mm. The thickness of the VB-EP substrate is 2 mm, and the bottom of the backscatter circuit is cover by a layer of printed silver film.

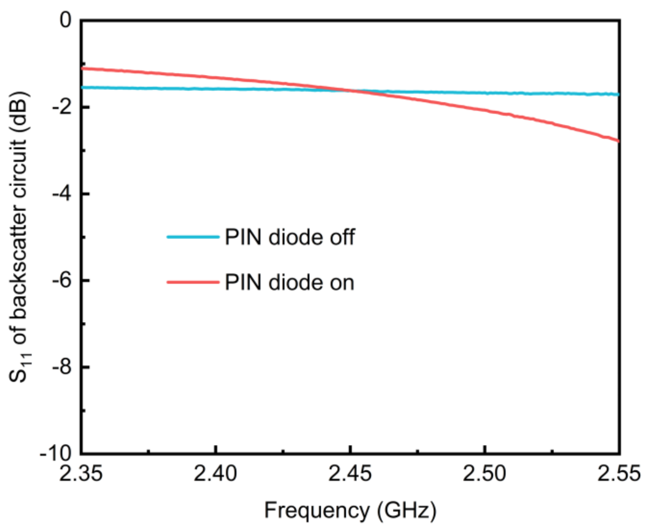

Fig. S18. The measured insertion loss of the transformative backscatter circuit.

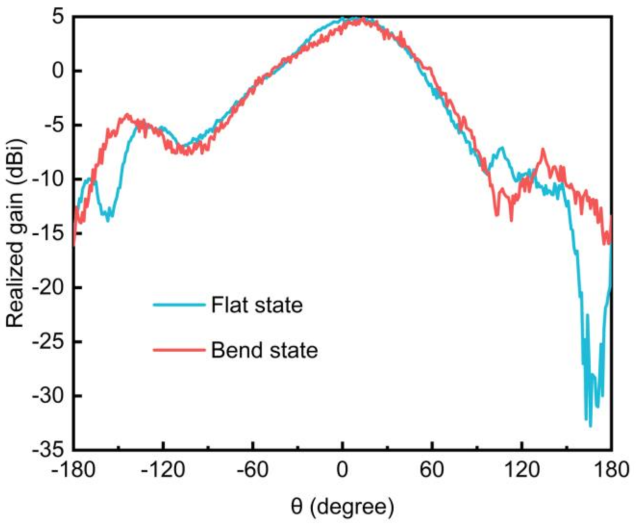

Fig. S19. Measured radiation patterns of the transformative 2.45 GHz antenna in bend state. The antenna was conformed to a cylindrical foam (diameter is 15 cm) and placed on the testing turntable in the anechoic chamber to obtain the radiation performance.

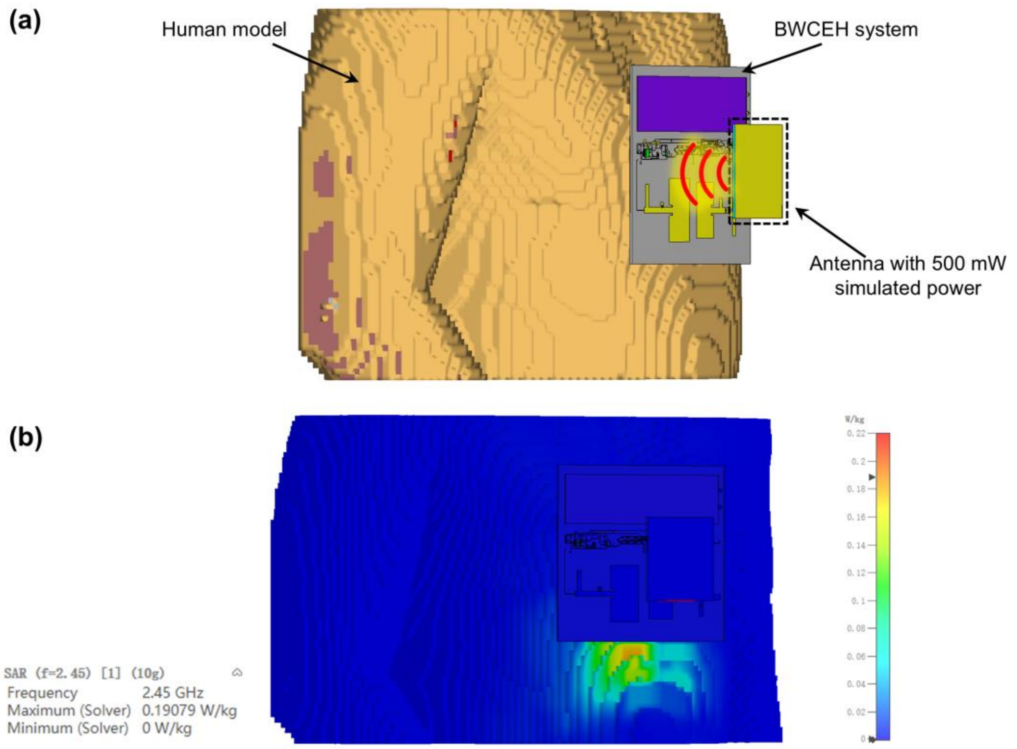

Fig. S20. SAR simulation of the BWCEH system. (a) The electromagnetic model of the BWCEH system and human tissue. (b) The SAR simulation result of the BWCEH system during backscatter communication.

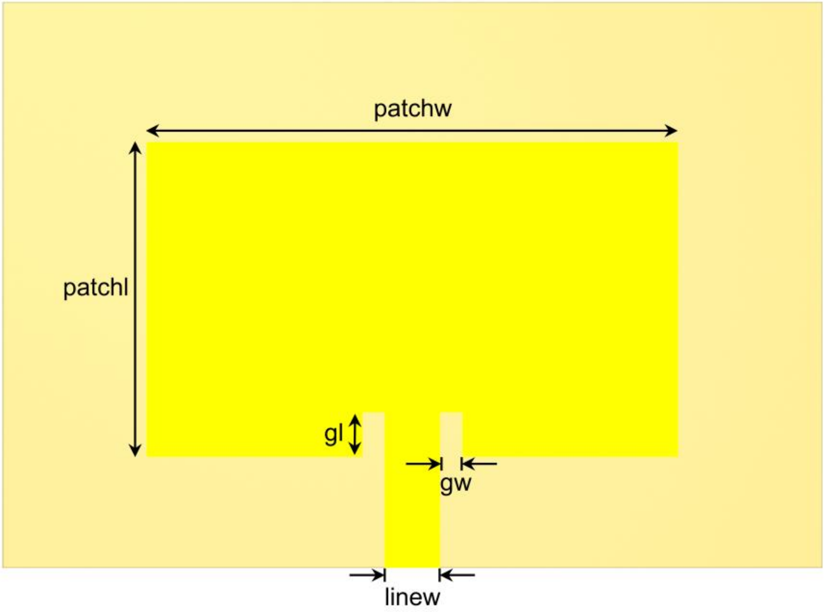

Fig. S21. Detailed geometric parameters of the 2.9 GHz transformative patch antenna which is responsible for RF energy harvesting. patchw = 48 mm, patchl = 28.4 mm, gw = 2 mm, gl = 4 mm, linew = 5 mm. The thickness of the VB-EP substrate is 2 mm, and the bottom of the backscatter circuit is cover by a layer of printed silver film.

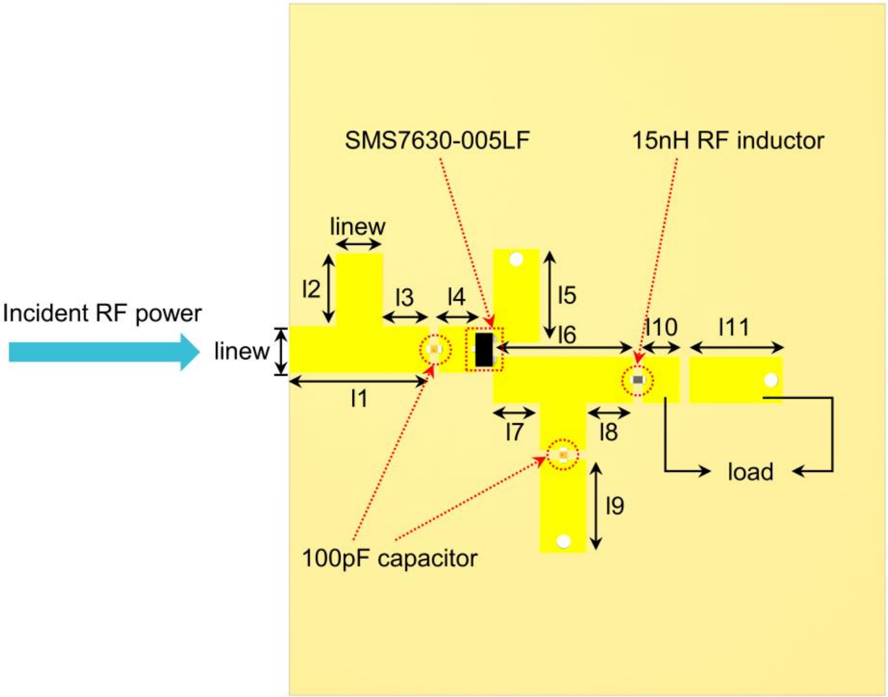

Fig. S22. Detailed geometric parameters of the 2.9 GHz transformative rectifying circuit which is responsible for converting RF energy to DC energy. l1 = 15 mm, l2 = 7.8 mm, l3 = 5 mm, l4 = 4 mm, l5 = 7.5 mm, l6 = 15 mm, l7 = 5 mm, l8 = 5 mm, l9 = 7.5 mm, l10 = 4 mm, l11 = 10 mm, linew = 5 mm. The thickness of the VB-EP substrate is 2 mm, and the bottom of the backscatter circuit is cover by a layer of printed silver film.

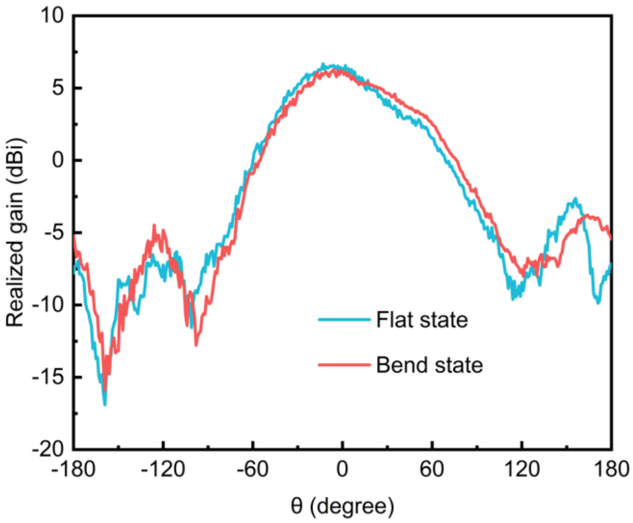

Fig. S23. Measured radiation patterns of the transformative 2.9 GHz antenna in bend state. The antenna was conformed to a cylindrical foam (diameter is 15 cm) and placed on the testing turntable in the anechoic chamber to obtain the radiation performance.

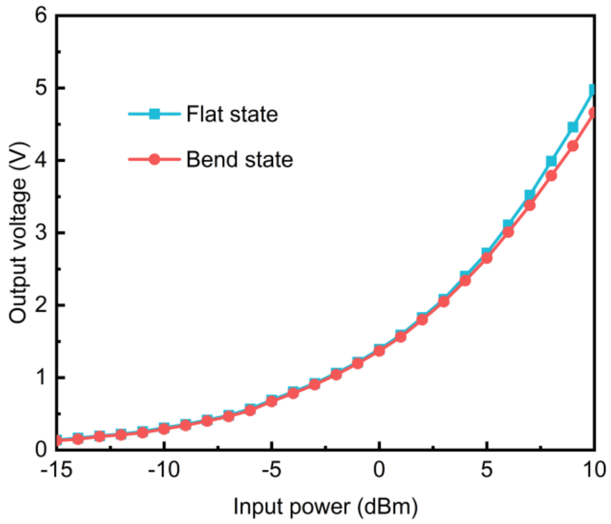

Fig. S24. Measured output voltage of the transformative rectifying circuit in bend state. The rectifying circuit was conformed to a cylindrical foam (diameter is 15 cm), and connected to a RF power source to measure the output voltage. In this test, the load of the rectifying circuit was connected to a 4 kΩ resistor.

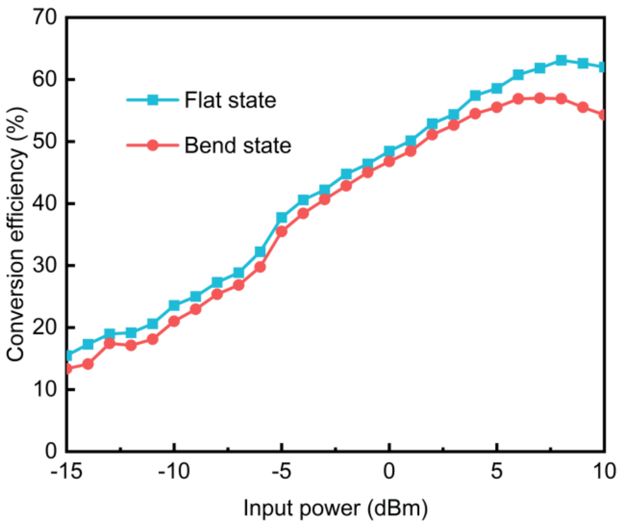

Fig. S25. Measured conversion efficiency of the transformative rectifying circuit in bend state. The rectifying circuit was conformed to a cylindrical foam (diameter is 15 cm), and connected to a RF power source to estimate the conversion efficiency. In this test, the load of the rectifying circuit was connected to a 4 kΩ resistor.

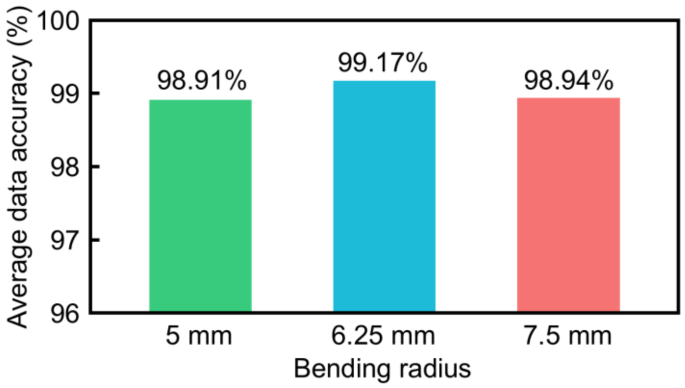

Fig. S26. The average wireless transmission accuracy of the system under different bending radius. The communication distance is 1.5 m, and the system is in the environment with 12000 lux sunlight.

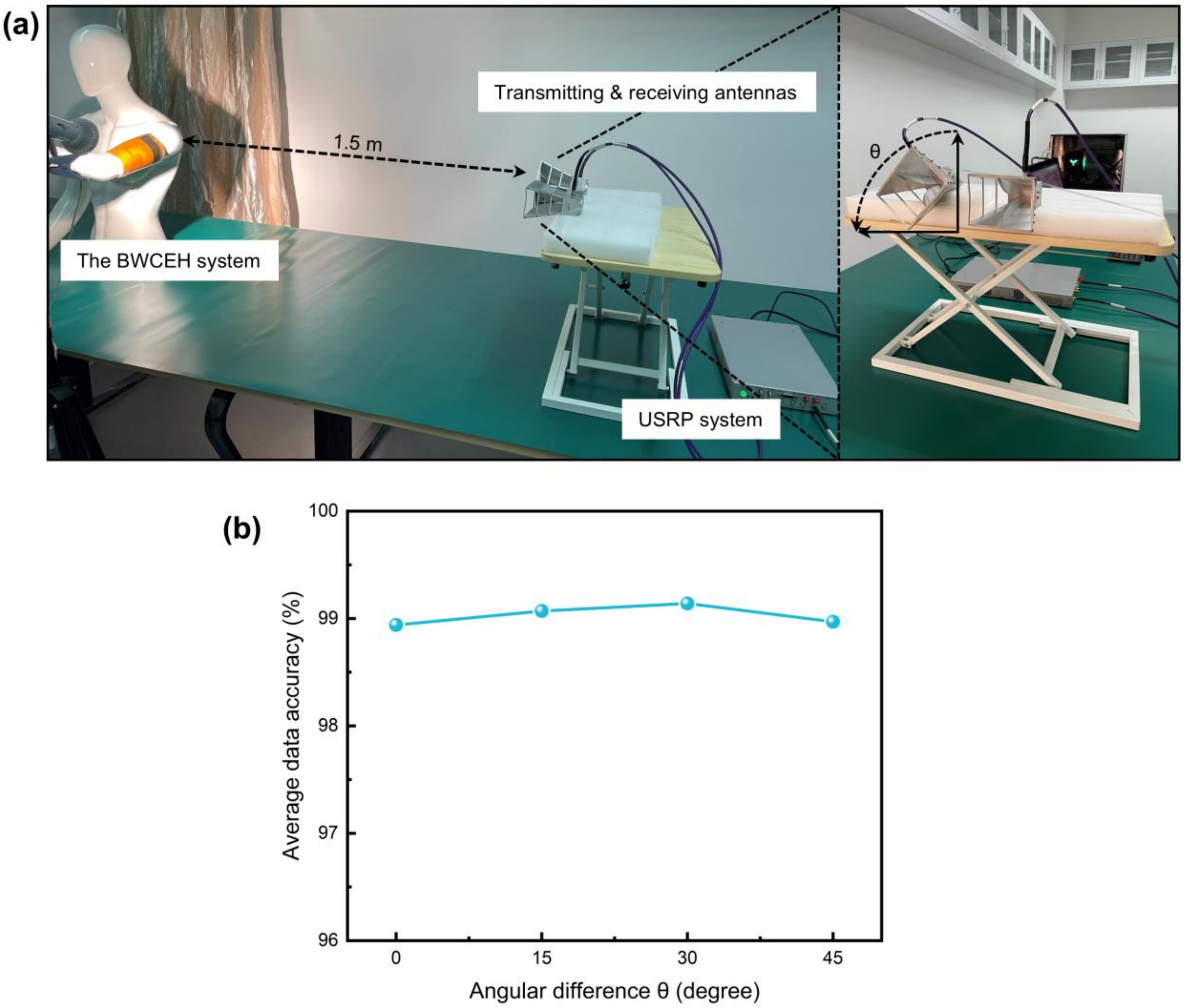

Fig. S27. The wireless communication experiment of the BWCEH system under polarization mismatching. (a) Experiment environment setup. (b) Average wireless transmission accuracy of the system under polarization mismatching.

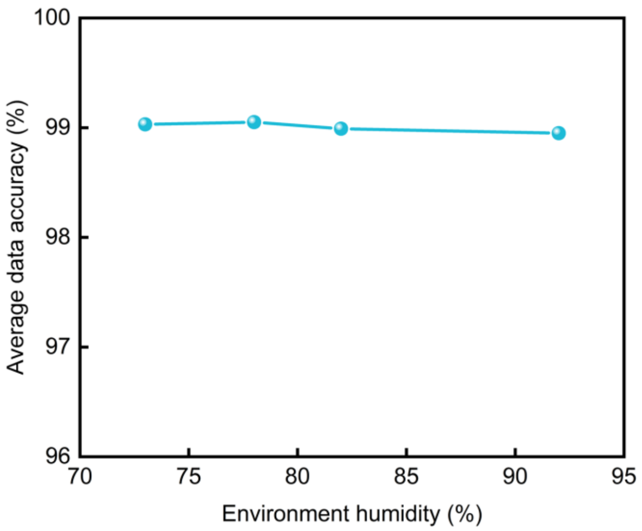

Fig. S28. Average wireless transmission accuracy of the BWCEH system in high-humidity environments. The communication distance is 1.5 m, and the system is in the environment with lamp light (2000 lux).

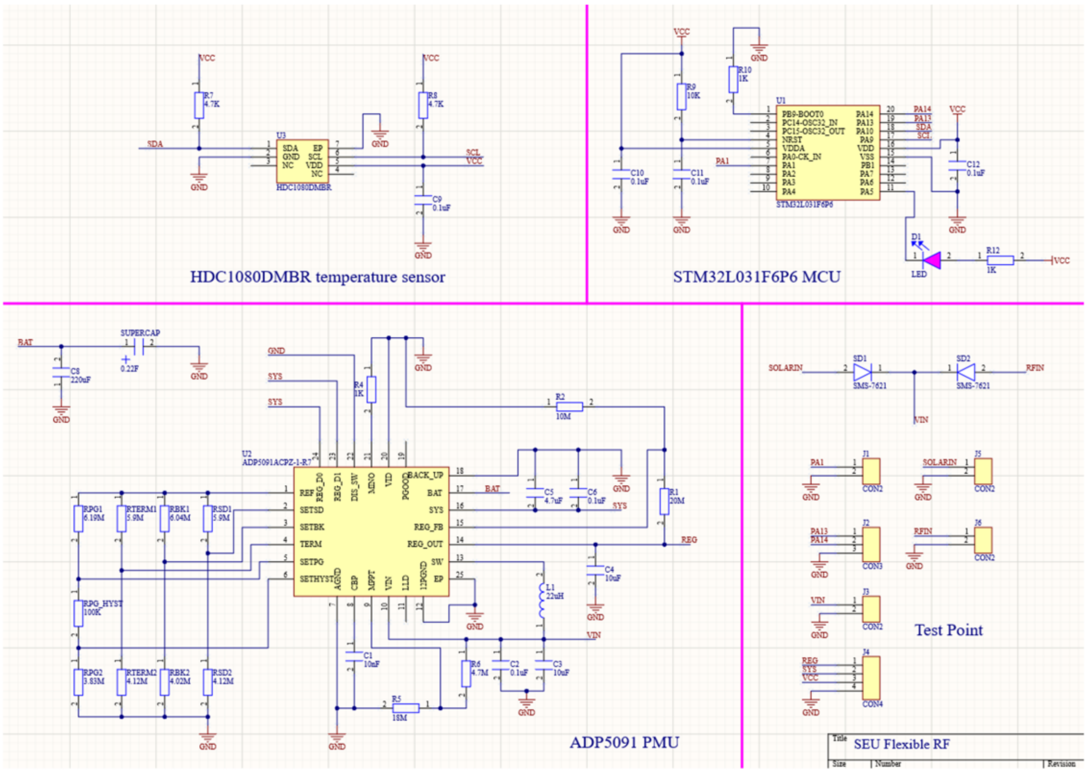

Fig. S29. Detailed schematic diagrams of the BWCEH system.

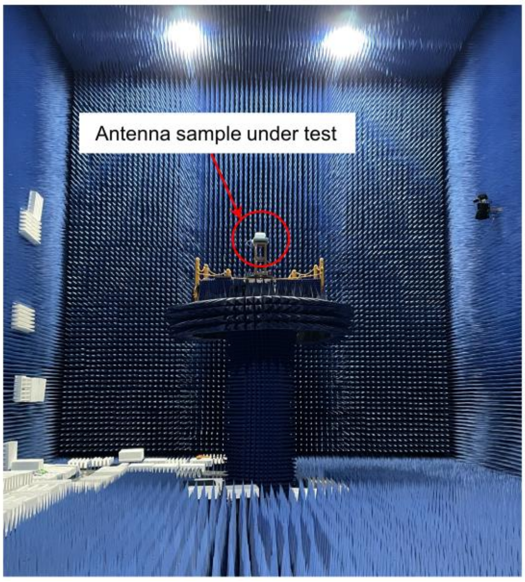

Fig. S30. Photograph of the anechoic chamber for radiation pattern measurement.
